# Lactic Acid Bacteria Fermentation and Endopeptidase Treatment Improve the Functional and Nutritional Features of *Arthrospira platensis*

**DOI:** 10.3389/fmicb.2021.744437

**Published:** 2021-12-08

**Authors:** Michela Verni, Cinzia Dingeo, Carlo Giuseppe Rizzello, Erica Pontonio

**Affiliations:** ^1^Department of Soil, Plant and Food Science, University of Bari Aldo Moro, Bari, Italy; ^2^Department of Environmental Biology, Sapienza University of Rome, Rome, Italy

**Keywords:** Spirulina, lactic acid fermentation, bioprocessing, *in vitro* protein digestibility, antioxidant activity, antimicrobial activity

## Abstract

This study aimed at investigating the effect of fermentation and enzymatic treatment on the degree of proteolysis of wet (WB), dried at low temperature (DB), and freeze-dried Spirulina (LB) proteins that affect the nutritional (e.g., amino acid content and profiles, and protein digestibility) and functional (e.g., antioxidant and antimicrobial activities) properties. The desiccation treatments influenced the unprocessed Spirulina characteristics because, compared with that in WB, peptides and free amino acids content was 73% lower in DB and 34% higher in LB. An integrated approach, including chromatographic and electrophoresis analyses, was used to evaluate the effect of the different bioprocessing options on protein profiles, release of peptides and amino acids, and the overall protein digestibility. Compared with the application of fermentation with the selected *Lactiplantibacillus plantarum* T0A10, the treatment with the endopeptidase Alcalase^®^, alone or combined, determined the most intense proteolysis. Moreover, the treatment with Alcalase^®^ of LB allowed the release of potentially bioactive compounds that are able to inhibit *Penicillium roqueforti* growth, whereas the combination of fermentation with *L. plantarum* T0A10 and Alcalase^®^ treatment increased Spirulina antioxidant properties, as determined by the scavenging activity toward ABTS radical (up to 60%) and antimicrobial activity against food pathogen *Escherichia coli*.

## Introduction

The challenge of feeding the growing world population and the pressure upon the global natural resources are forcing the food industry in developing alternative strategies to meat production, whose growing demand is ultimately not sustainable [World Health Organization (WHO), 2016]. Moreover, the consumption of functional foods and dietary supplements containing protein is an important and growing source of nutrition in diets of the consumers. Consumers use these products in their diet to achieve a healthy nutrition lifestyle (Kuesten and Hu, 2020). Apart from the source of energy (4 kcal/g protein) and essential amino acids, dietary proteins contain different sequences known as bioactive peptides (Sánchez and Vázquez, 2017) having the amino acid sequences with specific positive impacts on health. These are generally obtained by the proteolytic cleavage of a larger protein molecule through microbial fermentation, enzymatic activities, or during food processing (Patil et al., 2020). Protein hydrolyzates and peptides from natural resources can be used as “functional foods” and “nutraceuticals” on the basis of their bioactivity or as technological components due to their functional properties. The functional products and nutraceuticals may contain the whole hydrolyzate and/or isolated and purified peptides (Lafarga et al., 2016). Because the bioactivity and functionality of peptides depend on their amino acid composition, sequence, and molecular mass (Lassoued et al., 2015), peptides with varying effects might be derived from a single hydrolyzate.

Different microbes and algae, containing more than 30% protein in their biomasses, can provide an optimal balance of essential amino acids, moreover, requiring limited inputs, arable land, and water compared to plant-based protein production (Ritala et al., 2017).

Microbial protein sources, generally defined as single-cell protein (SCP), have high potential for the food and feed industry of developed and developing countries (Chama, 2019). Amidst SCP, Cyanobacteria, also known as blue-green algae, are a phylum of microorganisms related to bacteria but capable of performing photosynthesis; therefore, these are commonly included into the group of microalgae. Only few species of microalgae, among which *Arthrospira platensis*, *Chlorella vulgaris*, *Dunaliella salina*, and *Parietochloris incise*, are currently authorized for human consumption (Rizwan et al., 2018). Spirulina, which is the trade name referring to Cyanobacteria *A. platensis* and *Arthrospira maxima*, is one of the most well-known and worldwide cultivated microalgae (Lafarga et al., 2020). Spirulina is characterized by a very high protein content, which is around 60% on a dry weight basis and can reach up to 70% depending on cultivation conditions (Lafarga et al., 2020). The most relevant proteins in Spirulina are phycobiliproteins, water-soluble pigments that can be classified depending on their composition and chromophores content, with phycocyanins, phycoerythrins, and allophycocyanins being the main ones (Noreña-Caro and Benton, 2018). Phycobiliproteins have also been associated with several potential positive health effects, proving to be excellent sources of bioactive peptides with potential application in the functional foods industry as antihypertensive, antidiabetic, antiobesity, and antioxidant ingredients among other positive bioactivities assessed both *in vitro* and *in vivo* using animal models (Samarakoon and Jeon, 2012; Ovando et al., 2018; Lafarga et al., 2021). Aside from protein, Spirulina is also valued for the presence of several minerals, including potassium, calcium, magnesium, selenium, iron, zinc, and vitamins especially those belonging to the B group (Carcea et al., 2015; Lafarga et al., 2020).

Notwithstanding all the positive qualities Spirulina offers, some drawbacks, such as unusual color and flavor, limit its use as food ingredient (Chama, 2019). Moreover, the presence of cell wall, which, for many microalgae, is recalcitrant and not digestible by monogastric animals, limits the efficient extraction of intracellular components and prevents nutrients utilization, posing as the main nutritional issue (Coelho et al., 2020).

The aim of this study was to evaluate the effect of the combination of microbial and enzymatic treatments on the nutritional and functional features of Spirulina. Fermentation with selected strains of *Lactiplantibacillus plantarum* T0A10 already showing promising results in terms of bioactive peptides (Rizzello et al., 2017) and enzyme-assisted extraction method using Alcalase^®^ as proposed by Verdasco-Martín et al. (2019) were evaluated. Besides, aiming at evaluating the effect of drying on the nutritional quality, the above treatments were performed on wet, dried at low temperature, and lyophilized Spirulina biomasses. Indeed, different drying treatments are currently used at the industrial level to reduce the amount of water in Spirulina biomass, with some nutritional concerns related to the nutrient loss due to high temperature or removal of soluble compounds (Desmorieux and Hernandez, 2004). An integrated approach, including chromatographic and electrophoresis analysis, was used to evaluate the effect of the different bioprocessing options on the degree of proteolysis of Spirulina protein, which, in turn, may affect the digestibility and the release of potentially bioactive compounds.

## Materials and Methods

### Raw Material, Enzymes, and Microorganisms

Commercially available biomass of *Arthrospira platensis*, also known as *Spirulina platensis*, was purchased from Apuliakundi (Bitetto, Italy).

Alcalase^®^ 2.4 L FG (Novozymes, Denmark), a liquid commercial preparation of serine endopeptidase (EC. 3.4.21.62) obtained from *Bacillus licheniformis* (mainly subtilisin A; 27.3 kDa) that hydrolyzes amino esters including amino esters, was used in this study (Verdasco-Martín et al., 2019).

*Lactiplantibacillus plantarum* T0A10 (formerly classified as *Lactobacillus plantarum* T0A10), belonging to the Culture Collection of the Department of Soil, Plant and Food Sciences – Disspa (University of Bari, Italy), previously isolated from quinoa flour (Rizzello et al., 2016a) and selected based on the pro-technological features and metabolic traits affecting the functional properties of the matrix, was used in this study. The strain was routinely cultivated on De Man, Rogosa, and Sharpe (MRS) (Oxoid, Basingstoke, Hampshire, United Kingdom) until the late exponential phase of growth was reached (*circa* 10 h), as previously determined by the analysis of the kinetics of growth (Rizzello et al., 2016a). When used for fermentation, cells were harvested by centrifugation (10,000 *x g*, 10 min, 4°C), washed twice in 50 mM phosphate buffer (pH 7.0), and re-suspended in tap water at a density of 9 log10 cfu/ml.

### Pre-treatments and Bioprocessing of the Raw Material

Spirulina was provided from the supplier in two forms: as wet biomass (WB), obtained by separating cells from the culture medium by filtration (between 40 and 60 μm) and stored at −20°C until use; and the dry biomass (DB), obtained by pressing, extruding, and desiccating at low temperature (<40°C) fresh biomass.

WB had the following proximal composition: moisture, 80%; protein, 62.7% of dry matter (d.m.); carbohydrates, 18% of d.m.; fat, 6.1% of d.m.; total dietary fiber, 5.3% of d.m.; and ash, 6.4% of d.m.; whereas DB was characterized by the following composition: moisture, 12%; protein, 61.8% of d.m.; carbohydrates, 17.4%; fat, 7.2% of d.m.; total dietary fiber, 6.2% of d.m.; and ash, 5.9% of d.m.

An aliquot of WB, stored at −20°C, was freeze-dried (vacuum dehydration was carried out at −50°C) by using a Lio5P lyophilizer system (5Pascal, Trezzano sul Naviglio, Italy) (LB) and included in the study. LB had 5% moisture.

Aiming at improving Spirulina nutrients bio-accessibility and bioavailability, WB, DB, and LB were subjected to fermentation (F), enzymatic treatment with Alcalase^®^ (A), and combined Alacalase^®^/fermentation (AF) treatments. Before treatments and characterization, DB and LB were added with water reaching the same solid:liquid ratio of WB (*circa* 1:4 wt/wt).

To favor fermentation and Alcalase^®^ activity, the pH of all the biomass suspensions, characterized by a pH value of *circa* 9, was adjusted to 6.6 by using sodium acetate buffer (1 M, pH 5.2). Enzymatic treatment with Alcalase^®^ (1% vol/wt), was performed at 30°C for 24 h as reported by Verdasco-Martín et al. (2019), whereas fermentation was carried out with *L. plantarum* T0A10 (final cell density of *circa* 7 log10 cfu/g) at 30°C for 24 h.

A list of all samples and their respective abbreviations and the experimental design of the present study are provided in Supplementary Figure 1 and Supplementary Table 1, respectively.

### Microbiological and Biochemical Characterization

For microbiological analysis, 10 g of suspension was homogenized with 90 ml of sterile peptone water [1% (wt/vol) of peptone and 0.9% (wt/vol) of NaCl] solution. Presumptive lactic acid bacteria (LAB) were enumerated using MRS (Oxoid) agar medium supplemented with cycloheximide (0.1 g/L). Plates were incubated under anaerobiosis (AnaeroGen and AnaeroJar, Oxoid) at 30°C for 48 h. Cell densities of yeasts and molds were estimated on Yeast Peptone Dextrose Agar medium (Sigma-Merck, Darmstadt, Germany) supplemented with chloramphenicol (0.1 g/L), through pour and spread plate enumeration, respectively, and incubated at 25°C for 72 h. The yeast/mold differentiation was done by visual analysis of colony morphology. Total mesophilic aerobic bacteria (TMB) were determined on Plate Count Agar (Oxoid) at 30°C for 48 h, and total *Enterobacteriaceae* were determined on Violet Red Bile Glucose Agar (Oxoid) at 37°C for 24 h.

The values of pH were determined by a M.507 pHmeter (Crimson, Milan, Italy) equipped with a food penetration probe. Total titratable acidity was measured on 10 g of samples, which was homogenized with 90 ml of distilled water for 3 min in a Bag Mixer 400P (Interscience, St Nom, France), and expressed as the amount (milliliters) of 0.1 M NaOH to achieve the pH of 8.3. Organic acids were determined on water/salt-soluble extracts (WSEs) obtained from the biomass suspensions according to the method originally described by Osborne and modified by Weiss et al. (1993) and analyzed as reported by Pontonio et al. (2020).

### Proteins and Protein Derivatives Characterization

Aiming at assessing the effect of microbial or enzymatic activity on the Spirulina proteins, the biomass suspensions were centrifuged (10,000 *x g* for 10 min) to remove cell walls and insoluble residues and analyzed. Protein patterns were characterized by sodium dodecyl sulfate–polyacrylamide gel electrophoresis (SDS-PAGE) and two-dimensional electrophoresis (2DE), and peptide profiles and free amino acids (FAA), through liquid chromatography.

#### Protein and Peptides Concentration

Protein concentration in supernatant was estimated using the Bradford method (Bradford, 1976). For the analysis of peptides, supernatants (biomass suspensions centrifuged at 10,000 *x g* for 10 min) were treated with trifluoroacetic acid (0.05% wt/vol), centrifuged (10,000 *x g* for 10 min), and subject to dialysis (cutoff 500 Da) to remove proteins and FAA, respectively. Then, peptide concentration was determined by the *o*-phtaldialdehyde method as described by Church et al. (1983), and dialyzates were analyzed by reversed-phase fast-performance liquid chromatography (RP-FPLC).

#### Protein Patterns Characterization: Sodium Dodecyl Sulfate–Polyacrylamide Gel Electrophoresis and Two-Dimensional Electrophoresis

Aliquots of the samples containing *circa* 15 μg of protein were diluted 1:1 with sample buffer [0.125 M Tris-C1 (pH 6.8), 10% glycerol, 5% β-mercaptoethanol, and 2% SDS], treated at 100°C for 5 min, and analyzed by SDS-PAGE (Laemmli, 1970).

Two-dimensional electrophoresis was also carried out as described by Pontonio et al. (2020) using non-linear (from 4.0 to 7.0) gradient immobilized pH gradient strips (Amersham Pharmacia Biotech, Uppsala, Sweden) for the isoelectric focusing.

#### Peptide Profiles Characterization: Reversed-Phase Fast-Performance Liquid Chromatography

Peptides profiles were characterized by RP-FPLC, using a Resource RPC column and an ÄKTA FPLC equipment, with the UV detector operating at 214 nm (GE Healthcare Bio-Sciences AB, Uppsala, Sweden). Sample dialyzates containing peptides (1 mg/ml) were filtered with a 0.22-μm-pore-size filter and loaded onto the column. Gradient elution was performed at a flow rate of 1 ml/min using a mobile phase composed of water and acetonitrile (CH_3_CN), containing 0.05% trifluoracetic acid. The concentration of CH_3_CN was increased linearly from 5 to 46% between 16 and 62 min and from 46 to 100% between 62 and 72 min as described by Rizzello et al. (2010).

#### Free Amino Acids

For the analysis of the FAA, proteins and peptides in the samples were precipitated by addition of 5% (vol/vol) cold solid sulfosalicylic acid, holding the samples at 4°C for 1 h and centrifuging them at 15,000 *x g* for 15 min. The supernatant was filtered through a 0.22-μm-pore-size filter and diluted, when necessary, with lithium citrate (0.2 M, pH 2.2) loading buffer. FAA were analyzed by using a Biochrom 30^+^ series Automatic Amino Acid Analyzer (Biochrom Ltd., Cambridge Science Park, United Kingdom), equipped with a Li-cation-exchange column (internal diameter of 4.6 mm × 200 mm), using lithium citrate buffer eluents following the elution conditions recommended by the manufacturer. A mixture of amino acids at known concentrations (Sigma Chemical Co., Milan, Italy) was added with tryptophan (Trp), ornithine, and γ-aminobutyric acid and used as standard. Amino acids were post-column–derivatized with ninhydrin reagent and detected by absorbance at 440 (proline) or 570 (all the other amino acids) nm.

### *In vitro* Protein Digestibility

The *in vitro* protein digestibility (IVPD) was determined by the method proposed by Akeson and Stahmann (1964). The whole biomass suspensions were subjected to a sequential enzyme treatment, mimicking the *in vivo* digestion in the gastro-intestinal tract, and IVPD was expressed as the percentage of the total protein, which was solubilized after enzyme hydrolysis. The concentration of protein of digested and non-digested fractions was determined by the Bradford method (Bradford, 1976).

### Antioxidant Activities

The radical cation (2,2′-azino-di-[3-ethylbenzthiazoline sulfonate]) (ABTS+) scavenging capacity of all 12 samples was measured using the Antioxidant Assay Kit CSO790 (Sigma Chemical Co.), following the instruction of the manufacturer. Trolox (6-hydroxy 2,4,7,8-tetramethylchroman-2-carboxylic acid) was used as standard. The scavenging activity was expressed as Trolox equivalent.

### Antimicrobial Activity

The antifungal activity of Spirulina samples was assayed on the basis of hyphal radial growth rate of fungi (Quiroga et al., 2001). *Penicillium roqueforti* DPPMAF1, belonging to the Culture Collection Culture Collection of the Disspa (University of Bari, Italy) was used as indicator. *P. roqueforti* was used as the indicator microorganism for antifungal assays, because it corresponds to one of the most resistant fungi to chemical preservatives (Suhr and Nielsen, 2004). The biomass suspensions were centrifuged (10,000 *x g* for 10 min) and the supernatants sterilized by filtration on 0.22-μm membrane filters (Millipore Corporation, Bedford, MA, United States). Samples were added (10, 20, and 30% vol/vol, final concentration) to sterilized Potato Dextrose Agar, Oxoid (PDA). After mixing, aliquots of 20 ml were poured into Petri plates (diameter of 90 mm). Control plates contained PDA supplemented with 30% (vol/vol) of sterile water. The assay was carried out by placing a 3-mm-diameter plug of growing mycelia onto the center of Petri dishes containing the culture medium. Plates were incubated aerobically at 25°C. Three replicates were run simultaneously. The radial growth of mycelia (colony diameter, millimeters) in all plates was measured 6 days after inoculation. Each data point is the mean of at least four measurements of a growing colony. The percentage of growth inhibition was calculated from mean values as follows: percentage of inhibition = [(mycelial growth under control conditions - mycelial growth in the presence of WSE)/mycelial growth under control conditions] × 100.

Samples were further tested toward the potential gastrointestinal pathogen *Escherichia coli*, through the well diffusion assay, as described by Schillinger and Lücke (1989). *E. coli* DSM30083, belonging to the Culture Collection of the Disspa (University of Bari, Italy), was used as indicator strain. The assay was carried out using Luria Bertani medium (Oxoid Ltd.) soft agar medium (5 ml) overlaid on 15 ml of agar-H_2_O (2%, wt/vol). Indicator was inoculated at *circa* 4 log10 cfu/ml. Wells (diameter of 5 mm) were cut into the agar plates, and 50 μl of samples were placed in each well. Plates were stored at 4°C for 4 h to allow radial diffusion of the antimicrobial substance and incubated at 37°C for 24 h and before observation of the zones of inhibition. Fifty microliters of sterile water and chloramphenicol (final concentration 0.1 g/L) were used as negative and positive control, respectively. All the experiments were carried out in triplicate.

### Statistical Analysis

All the chemical and physical analyses were carried out in triplicate for each batch of Spirulina, either raw or bioprocessed. Data were subjected to one-way ANOVA; pair-comparison of treatment means was achieved by Tukey’s procedure at *P* < 0.05, using the statistical software Statistica 12.5 (StatSoft Inc., Tulsa, OK, United States). Data sets related to protein, peptide, and FAA concentration; IVPD; and antioxidant activity of biomasses on ABTS radical, antibacterial, and antifungal activities were analyzed through principal components analysis (PCA), using the software Statistica 12.5.

## Results

### Microbiological and Biochemical Characterization of Spirulina Biomasses

The microbiological and biochemical characterization of wet, dried, and lyophilized Spirulina is summarized in Table 1. Yeasts, molds, and *Enterobacteriaceae* were not detected in any sample, whereas differences were observed for mesophilic aerobic bacteria that increased (*circa* 3 log10 cfu/g), in both WB and DB, after incubation in presence of Alcalase^®^ (Table 1). When *L. plantarum* T0A10 was used as starter, alone or combined with the enzyme, the cell density reached up to 9.1 log10 cfu/g with an increase of *circa* two log cycles as compared to the initial inoculum. None of the microbial groups investigated were detected in lyophilized biomasses, except for presumptive LAB count in fermented samples (LB_*F*_ and LB_*AF*_).

**TABLE 1:** Microbiological and biochemical characteristics of wet (WB), dried at low temperature (DB), and lyophilized (LB) Spirulina biomass.

	TMB (log10 cfu/g)	LAB (log10 cfu/g)	Lactic acid (mmol/kg d.m.)	Acetic acid (mmol/kg d.m.)	Proteins (mg/g d.m.)	Peptides (mg/g d.m.)	TFAA (mg/g d.m.)
WB	<10 cfu/g*^c^*	<10 cfu/g*^c^*	n.d.	n.d.	180.02 ± 7.66*^a^*	59.3 ± 0.7*^e^*	77.21 ± 2.86*^f^*
WB_*A*_	3.6 ± 0.8*^b^*	3.5 ± 0.6*^b^*	1.8 ± 0.1*^c^*	n.d.	51.96 ± 2.52*^e^*	156 ± 20*^c^*	189.59 ± 7.88*^a^*
WB_*F*_	9.0 ± 0.5*^a^*	9.1 ± 0.7*^a^*	14.6 ± 0.6*^ab^*	7.7 ± 0.3*^b^*	150.90 ± 6.12*^b^*	76 ± 10*^d^*	136.07 ± 5.79*^b^*
WB_*AF*_	8.9 ± 0.7*^a^*	8.8 ± 0.5*^a^*	16.0 ± 0.6*^a^*	8.7 ± 0.4*^b^*	74.15 ± 4.45*^d^*	408 ± 30*^a^*	203.11 ± 10.01*^a^*
DB	<10 cfu/g*^c^*	<10 cfu/g*^c^*	n.d.	n.d.	182.65 ± 8.89*^a^*	21.1 ± 0.7*^f^*	16.30 ± 0.52*^i^*
DB_*A*_	3.5 ± 0.6*^b^*	3.5 ± 0.6*^b^*	n.d.	n.d.	113.96 ± 6.91*^c^*	86.0 ± 0.4*^d^*	52.86 ± 1.64*^g^*
DB_*F*_	9.0 ± 0.8*^a^*	8.9 ± 0.8*^a^*	12.9 ± 0.4*^b^*	6.1 ± 0.3*^c^*	113.68 ± 6.89*^c^*	105 ± 10*^d^*	19.11 ± 0.86*^i^*
DB_*AF*_	9.0 ± 0.6*^a^*	8.8 ± 0.7*^a^*	12.1 ± 0.5*^b^*	5.0 ± 0.2*^c^*	172.64 ± 6.02*^a^*	220 ± 17*^b^*	39.52 ± 4.48*^h^*
LB	<10 cfu/g*^c^*	<10 cfu/g*^c^*	n.d.	n.d.	162.89 ± 11.06*^b^*	74 ± 4*^d^*	109.62 ± 4.66*^d^*
LB_*A*_	3.5 ± 0.9*^b^*	3.4 ± 0.7*^b^*	n.d.	n.d.	50.96 ± 2.57*^e^*	81 ± 5*^d^*	122.54 ± 6.09*^cd^*
LB_*F*_	8.9 ± 0.8*^a^*	8.9 ± 0.9*^a^*	17.6 ± 0.8*^a^*	10.1 ± 0.5*^a^*	102.55 ± 10.89*^c^*	142 ± 2*^c^*	113.12 ± 5.41*^cd^*
LB_*AF*_	8.7 ± 0.6*^a^*	8.6 ± 0.7*^a^*	16.4 ± 0.6*^a^*	11.2 ± 0.6*^a^*	78.30 ± 3.11*^d^*	157 ± 3*^c^*	141.16 ± 5.66*^b^*

*TMB, total mesophilic bacteria; LAB, lactic acid bacteria; and TFAA, total free amino acids.*

*Data are the means of three independent experiments ± standard deviations (n = 3). ^a–i^Values in the same column with different superscript letters differ significantly (P < 0.05). n.d., not detected. All biomasses were also treated with Alcalase^®^ (A, 1% vol/wt), fermented with Lactiplantibacillus plantarum T0A10 (F, final cell density of circa 7 log10 cfu/g), or fermented and treated with Alcalase^®^ (AF). Enzymatic and microbial treatments were carried out at 30°C for 24 h.*

Because Spirulina cultivation occurs in alkaline environment, pH was adjusted to 6.6 to promote both microbial growth and Alcalase^®^ activity in biomass suspensions. A further pH drop was observed during incubation, especially when the selected strain was inoculated. DB_*F*_ and DB_*AF*_ showed the lowest pH values (5.25 ± 0.4 and 5.17 ± 0.7, respectively) (Table 1). As confirmed by the analysis of organic acids, significant amounts of lactic and acetic acids, which were not detected in not processed WB, DB, and LB, were found in fermented samples (Table 1). Except for WB_*A*_, containing a little amount of lactic acid, when Alcalase^®^ was singly used, organic acids were not detected.

Aiming at first assessing the overall effect of treatments on the organic nitrogen fraction, the concentration of proteins, peptides, and amino acids was evaluated. After removing the solid part of the suspensions, protein content of the three untreated samples was *circa* 180 mg/g, with values slightly lower in LB compared with that in WB and DB (Table 1). Significantly lower content of peptides and amino acids (up to 73%) were found in DB compared with that in WB, whereas they were both found at the highest concentration in LB (up to 34% higher than WB) (Table 1).

Significant (*P* < 0.05) changes were observed after bioprocessing. Protein content in WB_*A*_ was more than three times lower compared with that in WB, whereas peptides and total FAA (TFAA) were two and three times higher, respectively. All treated samples were characterized by lower protein content as compared with their corresponding untreated ones. Fermented samples (WB_*F*_ and WB_*AF*_) showed protein content from 18 to 50% higher than others. In turn, significant lower peptides and amino acids (from 28 to 592% and from 15 to 163%, respectively) concentrations were observed. The highest values were found in WB, where the combined effect of enzyme and fermentation allowed an increase of seven and three times of peptides and TFAA, respectively, reaching up to a total amount of 600 mg/g. A similar trend for proteins and peptides concentrations was observed in fermented samples of dried and lyophilized biomasses. However, compared to WBs, lower increases were observed in lyophilized Spirulina after treatments. A significant (*P* < 0.05) increase of the TFAA concentration was found only in LB_*AF*_, compared with that in LB (*circa* 110 mg/g).

### Protein Digestibility

**FIGURE 1:**
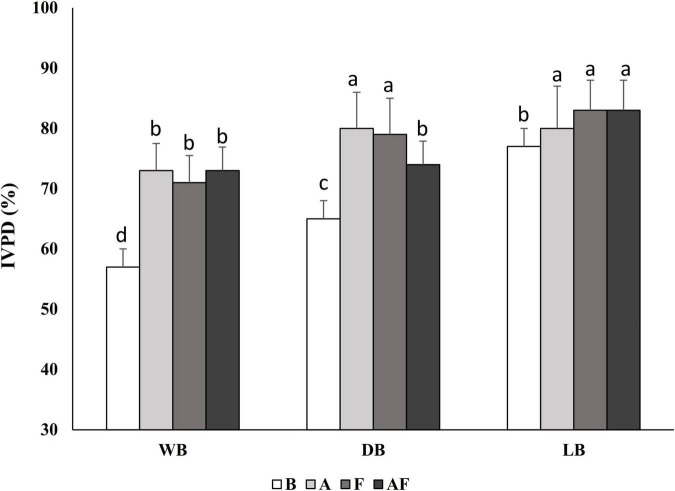
*In vitro* protein digestibility (IVPD) of wet (WB), dried at low temperature (DB), and lyophilized (LB) Spirulina biomass. All biomasses were also treated with Alcalase^®^ (A, 1% vol/wt), fermented with *Lactiplantibacillus plantarum* T0A10 (F, final cell density of *circa* 7 log10 cfu/g), or fermented and treated with Alcalase^®^ (AF). Enzymatic and microbial treatments were carried out at 30°C for 24 h.

Aiming at evaluating the effect of the different processing conditions on the susceptibility of the Spirulina protein fraction to digestive enzymes activity, the IVPD was determined by mimicking the gastro-intestinal digestion conditions. The IVPD of WB was 57% ± 4% and drying and lyophilization process affected it, leading to increases up to 20% in LB. According to the results reported in Figure 1, significant increases (*P* < 0.05) of the IVPD were also obtained through bioprocessing, especially in DB_*A*_, in which IVPD reached 80% ± 9%. Significant increases (*P* < 0.05), up to 16%, were observed in wet and dried biomasses treated with Alcalase^®^ and fermented (alone or combined).

### Effect of Treatments on Proteins and Protein Derivatives Patterns

#### Proteins Maps

**FIGURE 2:**
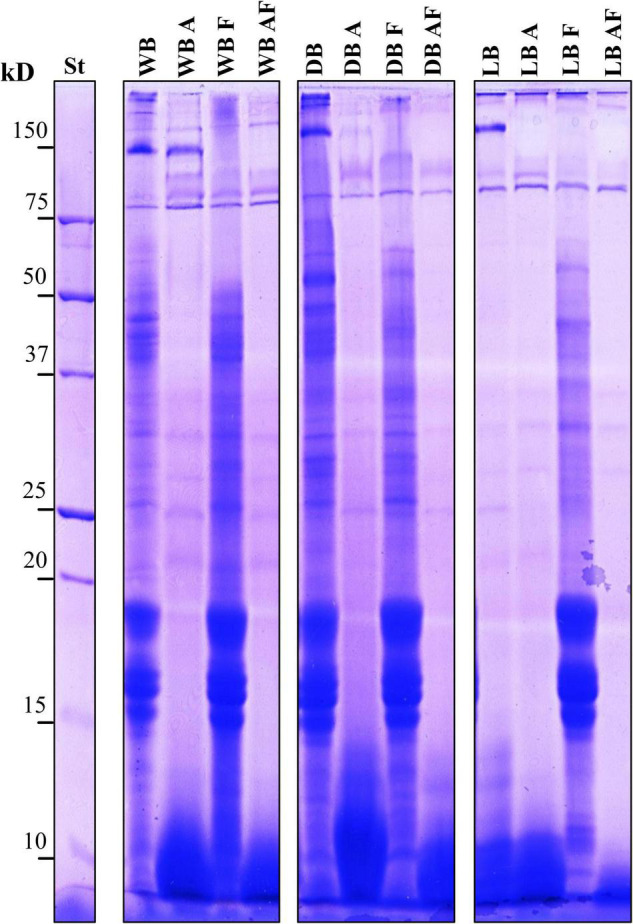
Sodium dodecyl sulfate–polyacrylamide gel electrophoresis (SDS-PAGE) patterns of total proteins of wet (WB), dried at low temperature (DB), and lyophilized (LB) Spirulina biomass. All biomasses were also treated with Alcalase^®^ (A, 1% vol/wt), fermented with *L. plantarum* T0A10 (F, final cell density of *circa* 7 log10 cfu/g), or fermented and treated with Alcalase^®^ (AF). Enzymatic and microbial treatments were carried out at 30°C for 24 h. St, protein standard (Bio-Rad, Hercules, CA, United States).

A preliminary view of the extent of the proteolysis in treated sample was given by SDS-PAGE protein patterns, as shown in Figure 2. WB was characterized by *circa* 30 bands, evenly distributed within the gel, of which those above 150 and between 15 and 20 kDa and between 37 and 50 kDa had the strongest intensity. A similar pattern to that found in WB was observed in DB, except for the higher intensity of few bands at *circa* 30 and 50 kDa. On the contrary, a much more different pattern was observed in lyophilized Spirulina, characterized by the disappearance of all the bands between 15 and 20 kDa and between 37 and 50 kDa. Samples treated with Alcalase^®^, both A and AF, exhibited a marked proteolysis which led to the complete disappearance of most of the bands in the range 15–75 kDa and to an intense presence of peptides below 15 kDa, forming a thick smear. In WB_*A*_ compared with WB, few more proteins were detected at *circa* 100 kDa, whereas in WB_*F*_ and WB_*AF*_, the bands right below 150 kDa disappeared. Moreover, a marked reduction of intensity of those bands that failed to enter the acrylamide knit was observed in A, F, and AF samples of wet and dried Spirulina (Figure 2). Lyophilized samples treated with Alcalase^®^, alone or combined to fermentation, compared to LB, lacked most of the bands over 100 kDa and were characterized by a lower intensity of those right below 15 kDa, especially in LB_*AF*_. In LB_*F*_, the bands between 15 and 20 kDa, which were not observed in unprocessed lyophilized Spirulina, had the strongest intensity. A band right over 75 kDa, instead, was not affected by any of the treatments in wet, dried, and lyophilized Spirulina (Figure 2).

As already highlighted by SDS-PAGE, some treatments affected the protein profile of Spirulina, with different magnitude according to the bioprocess used, observing significant variation of the total number and distribution of polypeptides in 2DE maps (Table 2). In detail, WB was characterized by 134 spots, mainly located in the middle section of the gel. In fact, 81 of the 134 had isoelectric point between 5 and 6, whereas, except for molecular weight (MW) higher than 75 kDa, the rest of the spots were evenly distributed among the other MW. Indeed, *circa* 30 spots were found in each of the MW ranges considered. In WB, the use of Alcalase^®^, alone or coupled with fermentation, led to the disappearance of all the spots except for two having pI between 4 and 5 kDa and *circa* 80 and 40 kDa. Fewer spots (24 less than WB) were observed in WB_*F*_, roughly maintaining the same distribution in terms of pI and MW. Nevertheless, proteolysis in WB_*F*_ caused the appearance of new polypeptides having MW <20 kDa and 5 < pI < 6. Compared to untreated WB, a very different profile was observed in dried Spirulina, mostly because of the drastically decrease (60%) of polypeptides having pI 5–6 and MW from 20 to 37 kDa. Moreover, 36 of the 64 spots had MW lower than 20 kDa. As for WB, the enzymatic treatment led to the hydrolysis of almost all proteins and polypeptides, while fermentation seemed to affect more polypeptides with 5 < pI < 6 and MW < 20 kDa. Overall, all processing applied led to a higher ratio of spot at pH 4–5 and 6–7, compared with their respective untreated biomasses WB and DB (Table 2). As for lyophilized Spirulina, 2DE maps of LB were much more compared with that of WB and DB. Only 26 small spots were detected, most of which with MW higher than 75 kDa and pI between 6 and 7. When treated with Alcalase^®^, especially when coupled with fermentation, the number of spots further decreased. On the contrary, fermentation alone determined an increase up to 52 spots, having mostly 4 < pI < 6 and MW between 25 and 75 kDa (Table 2).

**TABLE 2:** Estimated molecular weight range (kilodaltons) of polypeptides found in wet (WB), dried at low temperature (DB), and lyophilized (LB) Spirulina biomass.

	>75 kDa			37–75 kDa			25–37 kDa			20–25 kDa			<20 kDa		
	4–5	5–6	6–7	4–5	5–6	6–7	4–5	5–6	6–7	4–5	5–6	6–7	4–5	5–6	6–7
*Wet biomass*															
WB	1	–	–	1	28	5	4	22	9	7	13	9	5	18	12
WB_*A*_	1	–	–	1	–	–	–	–	–	–	–	–	–	–	1
WB_*F*_	1	–	–	1	18	4	4	18	7	3	13	8	4	21	8
WB_*AF*_	1	–	–	1	–	–	–	–	–	–	–	–	–	–	1
*Dried biomass*															
DB	1	–	–	1	5	2	3	8	–	2	3	3	8	16	12
DB_*A*_	1	–	–	1	–	–	–	–	–	1	–	–	–	1	–
DB_*F*_	1	–	–	1	3	–	5	1	–	1	1	–	2	4	10
DB_*AF*_	1	–	–	1	–	–	1	–	–	1	–	–	–	3	3
*Lyophilized biomass*															
LB	–	2	6	1	–	3	2	2	3	1	–	2	2	–	2
LB_*A*_	2	–	–	1	2	2	–	1	2	2	–	–	1	1	1
LB_*F*_	–	–	3	4	17	–	4	12	–	6	3	–	2	4	–
LB_*AF*_	–	–	2	3	–	–	1	–	–	–	–	–	–	1	1

*All biomasses were also treated with Alcalase^®^ (A, 1% vol/wt), fermented with Lactiplantibacillus plantarum T0A10 (F, final cell density of circa 7 log10 cfu/g), or fermented and treated with Alcalase^®^ (AF). Enzymatic and microbial treatments were carried out at 30°C for 24 h.*

#### Peptides Profiles

Aiming at understanding the modification occurred in Spirulina protein fraction, a complementary LC analysis of the peptides not detectable by the electrophoresis because it is not retained by the polyacrylamide gels (MW < 10 kDa) was carried out.

Lyophilized samples had the highest concentration of peptides, whereas in WB and DB, it was from three- to 10-fold lower (Supplementary Table 2). In dried biomasses (except for DB_*AF*_), although peptides concentrations significantly decreased (*P* < 0.05) in DB_*F*_ or remained the same in DB_*A*_, the number of peaks detected increased, if compared to unprocessed dried biomass. An opposite trend was observed in wet and lyophilized biomasses, where the increased concentration of peptides always corresponded to lower detected peaks (Supplementary Table 2). In all other processed samples, an increase of the hydrophilic peptides (in the eluent B gradient interval 0–46%) was observed.

#### Free Amino Acids

**FIGURE 3:**
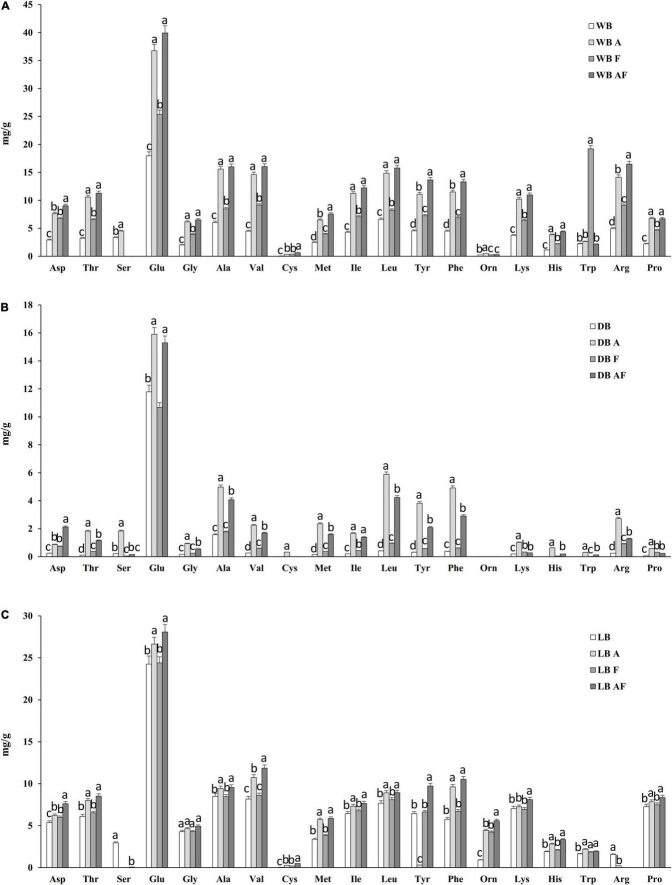
Free amino acids concentration (expressed as milligrams per gram) of wet (WB), dried at low temperature (DB), and lyophilized (LB) Spirulina biomass. All biomasses were also treated with Alcalase^®^ (A, 1% vol/wt), fermented with *L. plantarum* T0A10 (F, final cell density of circa 7 log10 cfu/g, or fermented and treated with Alcalase^®^ (AF). Enzymatic and microbial treatments were carried out at 30°C for 24 h. ^a–c^Values with different superscript letters differ significantly (*P* < 0.05).

The release of FAA is often a consequence of an intense proteolysis; hence, the evaluation of the treatments performed was concluded with the determination of the FAA. As reported in Figure 3, glutamic acid was the most abundant amino acid, reaching two-thirds of total amino acids in dried biomasses. An increment of all essential amino acids but Trp was observed in wet and dried biomasses. This increment resulted less pronounced in lyophilized Spirulina (A, F, and AF) because LB, compared with WB, had a concentration almost double. WB_*F*_ is the only sample in which a significant increase (*P* < 0.05) of Trp was observed, reaching 20 mg/g, whereas the highest increases and values for most FAA were observed in wet and dried biomasses treated with Alcalase^®^ and fermented (Figure 3). Cysteine (Cys) was the less represented amino acid, ranging from 0.0 to 0.06 mg/g, having DB and WB as the lowest and highest values, respectively. The treatment with Alcalase^®^ and fermentation, especially when coupled, allowed an increase of its content, up to 10 times the initial concentration in WB and LB. Whereas, in dried biomasses, only in DB_*A*_ Cys reached 0.32 ± 0.02 mg/g. Moreover, γ-aminobutyric acid, which was not detected in any of the biomasses prior treatment, was found in DB_*F*_ and DB_*AF*_ at 0.05 ± 0.00 and 0.12 ± 0.01 mg/g, respectively.

### Antioxidant Activity of Spirulina Biomasses

**FIGURE 4:**
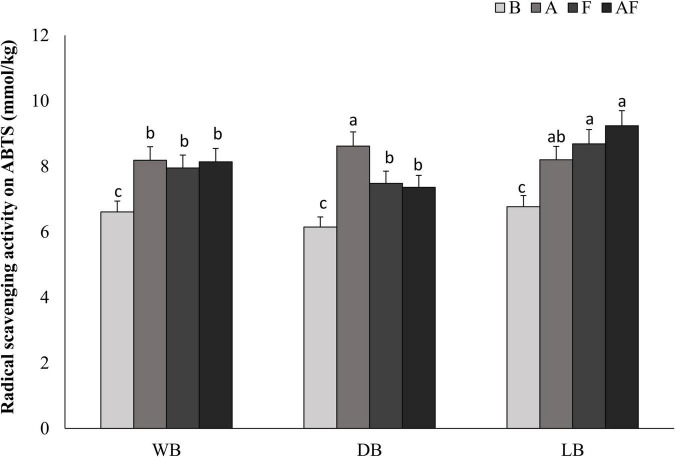
Antioxidant activity expressed as ABTS (millimoles per kilogram, Trolox) radical scavenging activity determined on wet (WB, panel **A**), dried at low temperature (DB, panel **B**), and lyophilized (LB, panel **C**). All biomasses were also treated with Alcalase^®^ (A, 1% vol/wt), fermented with *L. plantarum* T0A10 (F, final cell density of *circa* 7 log10 cfu/g), or fermented and treated with Alcalase^®^ (AF). Enzymatic and microbial treatments were carried out at 30°C for 24 h. ^a–c^Values with different superscript letters differ significantly (*P* < 0.05).

Besides phenols, the most attractive natural antioxidants mainly belong to the chemical class of peptides. Thus, biomasses, presumably containing these compounds, were subjected to the analysis of the radical scavenging activity (Figure 4). Antioxidant activity on ABTS radical ranged from 6.15 ± 0.05 to 9.24 ± 0.08 mmol Trolox eq./kg in biomasses. Significantly higher (*P* < 0.05) data were found in bioprocessed Spirulina as compared to the respective unprocessed, with increments up to 77%, with LB_*AF*_ having the highest values (9.24 ± 0.08 mmol Trolox eq./kg). Overall, the highest increases were found when fermentation with *L. plantarum* was coupled with Alcalase^®^, reaching up to 60%. In wet and dried biomasses, fermentation alone allowed increases (11 and 25%, respectively) significantly lower than all the other bioprocessed samples (Figure 4).

### Antimicrobial Properties of Spirulina Biomasses

Sterile extracts of Spirulina biomasses were added to PDA (10, 20, and 30%) to evaluate the inhibition of *P. roqueforti* growth (Table 3). A mild antifungal activity was observed in WB, especially at the highest concentration. Although, not always linear as the substitution level increased, overall higher values were found in lyophilized samples, compared with those obtained from wet and dried biomasses. In LB_*AF*_ and LB_*A*_, relevant increases in the antifungal activity were observed in comparison to the untreated lyophilized Spirulina (activity four- and sevenfold higher, respectively) (Table 3).

**TABLE 3:** Antimicrobial activity of wet (WB), dried at low temperature (DB), and lyophilized (LB) Spirulina biomasses.

	*Penicillium roqueforti*			*Escherichia coli*
	10%	20%	30%	
*Wet biomass*				
WB	0.0 ± 0.0*^f^*	12.9 ± 0.41*^e^*	13.2 ± 0.47*^e^*	+
WB_*A*_	13.4 ± 0.51*^d^*	12.8 ± 0.44*^e^*	13.1 ± 0.44*^e^*	++
WB_*F*_	13.5 ± 0.50*^d^*	13.0 ± 0.52*^e^*	13.3 ± 0.51*^e^*	+++
WB_*AF*_	13.5 ± 0.44*^d^*	13.1 ± 0.49*^e^*	30.4 ± 1.01*^c^*	+++
*Dried biomass*				
DB	4.3 ± 0.17*^e^*	13.0 ± 0.43*^e^*	21.7 ± 0.86*^d^*	−
DB_*A*_	26.1 ± 1.02*^c^*	21.7 ± 0.91*^d^*	21.6 ± 0.88*^d^*	±
DB_*F*_	13.0 ± 0.49*^d^*	13.2 ± 0.43*^e^*	13.2 ± 0.54*^e^*	+
DB_*AF*_	13.6 ± 0.46*^d^*	13.4 ± 0.41*^e^*	13.1 ± 0.53*^e^*	±
*Lyophilized biomass*				
LB	13.1 ± 0.51*^d^*	26.2 ± 0.84*^c^*	8.7 ± 0.23*^f^*	±
LB_*A*_	34.8 ± 1.32*^b^*	34.8 ± 1.36*^b^*	100.0 ± 2.29*^a^*	+
LB_*F*_	34.7 ± 1.36*^b^*	21.9 ± 0.78*^d^*	34.8 ± 1.09*^c^*	+++
LB_*AF*_	43.5 ± 1.71*^a^*	47.8 ± 1.03*^a^*	56.5 ± 1.18*^b^*	+++

*Data are the means of three independent analyses. ^a–f^Values in the same column with different superscript letters differ significantly (P < 0.05). All biomasses were also treated with Alcalase^®^ (A, 1% vol/wt), fermented with Lactiplantibacillus plantarum T0A10 (F, final cell density of circa 7 log10 cfu/g), or fermented and treated with Alcalase^®^ (AF). Enzymatic and microbial treatments were carried out at 30°C for 24 h. Antifungal activity was determined by hyphal radial growth inhibition of Penicillium roqueforti DPPMAF1 after 6 days of incubation at 25°C in PDA containing 30% vol/vol of the extract. Inhibitory activity against gastrointestinal pathogen Escherichia coli was scored as follows: −, no inhibition; ±, inhibition halo of 1–2 mm; +, inhibition halo of 3–4 mm; ++, inhibition halo of 5–6 mm; +++, inhibition halo of 7–9 mm.*

The antimicrobial potential of Spirulina biomasses against *E. coli* was also assessed. A weak inhibition of the indicator strain (inhibition halo of *circa* 3 mm) was observed in wet and lyophilized biomasses; however, no activity was detected for DB. When fermentation was used in wet and lyophilized Spirulina, alone or coupled with Alcalase^®^, inhibition halos ranged from 7 to 9 mm. Whereas, no significant differences were observed in dried biomass after treatments (Table 3).

## Discussion

Nowadays, the nutritional composition – particularly rich in proteins, minerals, vitamins, and essential fatty acids along with the evolving awareness of diet influence on the state of health and the pressing need of sustainable food sources – is the main reason to its fast-emerging applications as food supplement and ingredient. Microalgae feasibility as food ingredients or source of nutrients and/or bioactive compounds and their health effects have been widely studied (Barros de Medeiros et al., 2021). Indeed, as bioactive peptides are generated from proteins and microalgae are protein-rich feedstocks, the potential utilization of microalgae as a source for peptides has gained increased interest during the past decade (Lafarga et al., 2021). Currently, only few species of microalgae are grown, produced, and used on a commercial scale, and among all, *Arthrospira platensis* is one of the most used (Barros de Medeiros et al., 2021). Overall, the market value of algae products, which in 2018 amounted to 2.6 billion US$, is forecast to reach over 3.45 billion US$ by 2025 (Statista, 2019).

Fresh Spirulina, in its natural state, is made of regularly wrapped spiral multicellular filaments, which are well compartmentalized with a lot of proteins partitioned within the cells. However, the cell wall is made of multiple layers, the first of which containing β-1,2-glucan (Vernès et al., 2019; Chen et al., 2020), that might limit the bioaccessibility of such acclaimed components.

In this framework, the present study aimed at optimizing a biotechnological protocol able to favor the proteolysis of the Spirulina proteins, which may increase the functional (e.g., bioactive compounds) and nutritional (e.g., protein digestibility) features, by comparing and combining two methods recently explored by the literature: treatment with Alcalase^®^, having endopeptidase activity; and fermentation with a selected strain of LAB. Among bioactive compounds used as food ingredients, peptides are one of the most studied (Fernandes et al., 2019). Although peptides can be released using several treatments, the use of commercial proteases is the most common strategy because of the high specificity of proteins and the possibility to control the process (Lafarga et al., 2021). Among other enzymatic preparations, Alcalase^®^ was found to increase the extraction yield of hydrophilic compounds by 90% compared to the simple solvent extraction (Verdasco-Martín et al., 2019). Moreover, the Alcalase^®^ treatment contributed to the production of bioactive peptides, which can positively impact human and animal metabolism and health, as extensively reported by authors (Tacias-Pascacio et al., 2020; Lafarga et al., 2021). On the other hand, microbial fermentation has widely been reported as a promising tool for the release of bioactive peptides with *Lactobacillus* species being the predominantly used (Patil et al., 2020). Fermentation with selected strains of *L. plantarum* was attempted by de Marco Castro et al. (2019) and Niccolai et al. (2019) to increase *in vitro* antioxidant properties. Nevertheless, because the functionality of protein hydrolyzates may differ between strains due to the presence of different proteolytic systems in microorganisms (Patil et al., 2020), the specific *L. plantarum* T0A10 strain, previously selected to release bioactive compounds (Rizzello et al., 2017), was used in this study.

Because of its high-water content and, therefore, high perishability, drying processes are necessary to preserve Spirulina, although often associated with loss of quality compared to the fresh product, especially if conducted at high temperatures (Desmorieux and Hernandez, 2004). Although spray drying is preferred at industrial level, small Spirulina producers generally dry the produced biomass using convective drying processes. Other strategies such freeze-drying have been evaluated but are not yet as relevant because considered a time- and energy-consuming manufacturing process (Lafarga et al., 2021). Nevertheless, freeze drying is considered as the best way to preserve the high-value compounds (Soni et al., 2017; Noreña-Caro and Benton, 2018), and several freeze-dryers and continuous freeze-drying tunnels have already been used for the food industry (Pisano et al., 2019). An *ad hoc* optimization of the parameters may help resolve scale-up issues by reducing cost and operation time (Butreddy et al., 2020).

In this study, wet (80% moisture), dried (12% moisture), and freeze-dried (5% moisture) Spirulina were used and subjected to the enzymatic treatments and or microbial fermentation.

The analysis of the three different types of Spirulina biomass showed remarkable lower contents of peptides and FAA in dried compared to WB. The process used for drying Spirulina after harvesting includes biomass filtration and pressing to drain excess water, extrusion in 1- to 2-mm thin layers or rods (“spaghetti” shape), and drying at relatively low temperature. A large part of the soluble compounds like amino acids and peptides are therefore lost, explaining the gap between their content in wet and dried biomasses (Table 1). Moreover, the decrease of protein (10–20%) during drying was previously reported (Oliveira et al., 2009) with losses directly proportional to the process temperature (Desmorieux and Hernandez, 2004). Overall, drying treatments are also responsible for an alteration of the biomass, filaments unfold, and begin to fragment; nevertheless, the cells remain intact (Vernès et al., 2019).

On the contrary, increases of peptides and amino acids (up to 180 mg/g combined) were observed in lyophilized compared to WB. In this case, Spirulina was stored at *circa*−20°C after harvesting, until the freeze-drying. Low temperature storage is considered as the optimal approach for cryopreservation of organisms, yet it relies upon their ability of being cooled avoiding cryo-injury induced by physical and chemical changes associated with freezing. Kapoore et al. (2019) observed that storage at −15°C, especially if for prolonged time (up to 4 months), leads to a rapid reduction in viability of *C. vulgaris* due to damage associated with ice crystal development and colligative wound due to excessive concentrations of solutes (Mazur, 1965). The concentration of solutes can affect the permeability of the membrane and, as a result, large quantities of low MW solutes dialyze, in quantity roughly proportional to the percentage of damaged cells (Mazur, 1965). It is therefore possible that the freeze-storage and freeze-drying processes led to the lysis, or at least damage, of the cells, causing the release of peptides and amino acids. Indeed, the freezing and drying steps of the lyophilization process may cause protein unfolding and alteration of the protein structure due to the dehydration stress and ice-liquid interfacial stress (Butreddy et al., 2020).

Spirulina biomass is normally cultivated in medium varying for their osmotic, temperature, and salt concentrations, mostly being highly alkaline (Lafarga et al., 2021); indeed, the pH of WB exceeded 9, preventing both enzymatic and microbial activity. Thus, a solution of food grade sodium acetate was added to all samples to decrease the pH to *circa* 6.6, and it remained stable throughout the treatments except when *L. plantarum* was inoculated. The high pH during cultivation also prevents the growth of other microbial species into the substrate (Soni et al., 2017). Indeed, except for mesophilic aerobic bacteria, none of the microbial groups investigated (yeasts, molds, and *Enterobacteriaceae*) were found in raw and treated biomasses. However, the incubation at 30°C for 24 h with Alcalase^®^ allowed an increase of total mesophilic bacteria, probably due to the higher availability of nutritional compounds. As expected, when inoculated, LAB exceeded a cell density of 9 log10 cfu/g, leading to the synthesis of high concentrations of organic acids.

A more comprehensive overview of what the protein fraction underwent to during the treatments was provided by electrophoresis. The protein fraction in Spirulina mainly consists of phycobiliproteins, chromophore-bearing, highly fluorescent, and water-soluble protein components of the photosynthetic complexes of cyanobacteria. They are classified into two groups on the basis of their colors: red for phycoerythrin and blue for phycocyanin. The latter includes C-phycocyanin (C-PC), R-phycocyanin (R-PC), and allophycocyanin (APC) (Kuddus et al., 2013). APC and C-PC often occur as a trimer, hexamers, and other oligomers, and C-PC constitutes up to 20% of the dry weight of Spirulina (Chaiklahan et al., 2011; Kuddus et al., 2013). SDS-PAGE results showed an intense proteolysis determined by Alcalase^®^, involving mainly the α- and β-subunits of phycobiliproteins, having MW slightly lower than 20 kDa (Chaiklahan et al., 2011). Because of the enzymatic treatment, as well as to the proteolytic system of *L. plantarum* T0A10, the other major change in the protein profile concerned proteins with MW over 100 kDa, which faded considerably in biomasses singly and combined treated with Alcalase^®^ and microbial strain. Moreover, lyophilized biomass was characterized by much weaker bands, compared to WB, with the loss of polypeptides between 15 and 20 kDa (Figure 2). It is hypothesized that the precipitation of some proteins followed the salt concentration increase due to the freeze-drying. With water removal, ions and proteins compete for water molecules, leading to protein-protein interactions, which become stronger than protein-water interaction, resulting in aggregation and precipitation (Wingfield, 2016).

Because mono- and bi-dimensional electrophoresis only deemed proteins within the range 10–200 kDa, the effect of the treatments on peptides below 10 kDa and amino acids was evaluated through chromatographic techniques. The chromatographic analysis based on hydrophobic interactions highlighted significant differences in the peptide fraction among samples. Although the total number of detected peaks decreased with bioprocessing (compared to wet and lyophilized biomasses), the total area was overall subjected to significant increases. Indeed, values 70, 36, 30, and 50% higher than their corresponding untreated samples were found for wet, dried, and lyophilized biomasses treated with Alcalase^®^ and *L. plantarum* T0A10, and lyophilized biomasses treated with Alcalase^®^, respectively (Supplementary Table 2), suggesting substantial degradation of higher MW proteins, as confirmed by the SDS-PAGE and 2DE. As a matter of fact, because of the wide range of amino acids that it can recognize, the hydrolysis catalyzed by Alcalase^®^ has a strong tendency to give hydrolyzates with many peptides of small size (Tacias-Pascacio et al., 2020). Whereas, the contribution of the complex system of proteases and peptidases own by LAB is well known (Kieliszek et al., 2021).

A further confirmation, of what observed so far, was provided by analyzing the single FAA profiles. Their content in wet Spirulina was in accordance with that reported elsewhere (Mišurcová et al., 2014; Bashir et al., 2016). Spirulina contains lysine and sulfur containing amino acids (further enhanced by Alcalase^®^ and fermentation), which are generally limiting in cereals; thus, it can be an excellent choice for supplementation of cereals-based products. Whereas in wet and lyophilized biomasses, A and AF were the treatments that led to the highest releases of FAA, linearly with what reported for peptides and proteins, in dried Spirulina, Alcalase^®^ allowed the most relevant increases, up to 24-fold higher than the initial concentration (Figure 3). Notwithstanding, it is worth of notice that the amount of each FAA in dried biomass, due to drying process explained above was one to 21 times lower than WB, with many amino acids close to 0 mg/g.

The effect of the treatments on protein digestibility was evaluated by mimicking the gastrointestinal digestion with an *in vitro* multi-step enzymatic assay. Digestibility of WB was similar to that previously reported (Kose et al., 2017) and substantial increases were found in lyophilized Spirulina, as consequence of the release of peptides and FAA. Overall, it was found that Alcalase^®^ and fermentation (alone or combined) led to IVPD increases proportional to the proteolysis degree, compared with their respective untreated samples.

Besides the increased digestibility of the protein fraction, proteolysis, carried out by Alcalase^®^ or LAB, can result in the formation of bioactive peptides having any number of functions (Rizzello et al., 2016b; Tacias-Pascacio et al., 2020). Moreover, bioactive peptides derived from Spirulina were already characterized for antibacterial, antiallergic, antihypertensive, antitumor, and immunomodulatory properties (Ovando et al., 2018; Lafarga et al., 2021). Hence, all samples were tested for their ability to inhibit microbial growth and scavenge radicals. In general, biomass suspensions had high antioxidant activity, suggesting a higher contribution of the protein component of the matrix, to the antioxidant properties observed. Alcalase^®^ and fermentation were the treatments that led to the highest increases of the radical scavenging activity, obtaining similar results to those reported by Niccolai et al. (2019) when Spirulina was fermented with a *L. plantarum* strain.

One of the main concerns of the food industry is ensuring food safety during shelf life, preventing contamination by pathogenic and spoilage microorganisms, and the use of natural antimicrobic instead of synthetic ones is becoming the preferred option. In our study, 100% inhibition of *P. roqueforti* growth was achieved when lyophilized biomass enzymatically treated was used. As a matter of fact, antimicrobial activity of protein hydrolyzates obtained with Alcalase^®^ was reported (Tacias-Pascacio et al., 2020). On the other hand, only fermented Spirulina (F, AF, and SF) was able to inhibit food pathogen *E. coli*, but most of all, only in wet and lyophilized Spirulina. Much more subtle differences were observed in dried biomasses, most likely because the compounds responsible for such activity derive from the hydrolysis of soluble proteins lost during the drying process. However, further studies are necessary to evaluate a higher inhibition spectrum against fungi and food pathogenic bacteria (including gram positive bacteria), as well as the compounds responsible for such activity.

**FIGURE 5:**
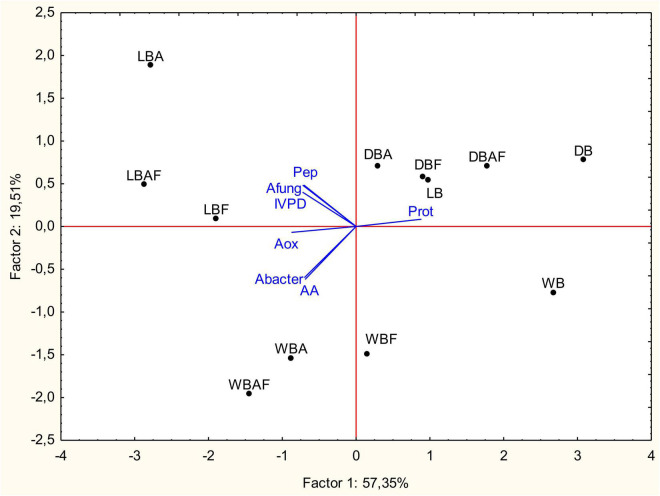
Principal components analysis (PCA) based on the biochemical, nutritional, and functional characteristics, and inhibitory activity of wet (WB), dried at low temperature (DB), and lyophilized (LB) Spirulina biomass. All biomasses were also treated with Alcalase^®^ (A, 1% vol/wt), fermented with *L. plantarum* T0A10 (F, final cell density of *circa* 7 log10 cfu/g), or fermented and treated with Alcalase^®^ (AF). Enzymatic and microbial treatments were carried out at 30°C for 24 h. Prot, protein concentration; Pep, peptide concentration; AA, amino acid concentration; IVPD, *in vitro* protein digestibility; Aox_*ABTS*_, antioxidant activity of biomasses on ABTS radical; A_*bacter*_, antibacterial activity; A_*fung*_, antifungal activity.

Data collected from the characterization of Spirulina biomasses were subjected to PCA as showed in Figure 5. The first and second factors explained, respectively, the 57.35 and 19.51% of the total variance. Except for dried samples, the first factor separated bioprocessed (A, F, and AF) from raw samples, whereas the second factor clearly distinguished wet and lyophilized biomasses. Bioprocessed samples of wet and lyophilized biomasses were characterized by the lower protein content to which corresponded higher concentration of peptides and FAA with potentially bioactive properties. On the contrary, more subtle changes were apported by enzymatic treatment and fermentation, when performed on dried biomasses, hence the concentration in the I quarter of the PCA plane (Figure 5).

## Conclusion

This study aimed at assessing the effect of desiccation and biotechnological treatments on the Spirulina protein profile. Overall, aiming at stabilizing the biomass, by reducing the moisture content, the lyophilization proved to be the most promising method due to its effect on the structure of Spirulina, which led to the release of amino acids and small peptides in substantial amount. On the other end, exsiccation at low temperature, because of the filtering and pressing process, resulted in the loss of most of the soluble compounds originally characterizing Spirulina biomasses. Hence, more thought should be put when considering the drying method to apply and, although lyophilization entails expenses seldom accepted at industrial level, the cost-benefit ratio represents the needle of the scale.

Moreover, to enhance the bioaccessibility of peptides and amino acids, while releasing potentially bioactive compounds showing antioxidant and antimicrobial activities, the combination of Alcalase^®^ and fermentation with selected *L. plantarum* T0A10 was found to be the most appropriate approach. The release of bioactive peptides outside the gastro-intestinal tract is of major significance because they not only confer nutritional advantages to the body once ingested but also to the food itself, preventing or delaying its spoilage and shelf life.

Overall, freeze-dried Spirulina biomass treated with Alcalase^®^ and fermented with selected *L. plantarum* T0A10 showed scavenging activity toward ABTS radical (up to 60%) and promising antimicrobial activity against fungal spoilage *P. roqueforti* and food pathogen *E. coli*. Therefore, on the basis of the results of this work, the selected treatments will be the topic of further studies involving the identification of the compounds responsible for such activities and the use of bioprocessed Spirulina in the development of functional foods.

## Data Availability Statement

The original contributions presented in the study are included in the article/Supplementary Material, further inquiries can be directed to the corresponding author/s.

## Author Contributions

MV was responsible for formal analysis, data curation, investigation, and writing the original draft. CD was responsible for formal analysis and data curation. CR was responsible for resources and funding acquisition and for manuscript revision. EP was responsible for the conceptualization of the study, investigation, validation, writing, reviewing, and editing. All authors contributed to the article and approved the submitted version.

## Conflict of Interest

The authors declare that the research was conducted in the absence of any commercial or financial relationships that could be construed as a potential conflict of interest.

## Publisher’s Note

All claims expressed in this article are solely those of the authors and do not necessarily represent those of their affiliated organizations, or those of the publisher, the editors and the reviewers. Any product that may be evaluated in this article, or claim that may be made by its manufacturer, is not guaranteed or endorsed by the publisher.
