# Supplementary material for: Lactic Acid Bacteria Fermentation and Endopeptidase Treatment Improve the Functional and Nutritional Features of Arthrospira platensis
Source: Front Microbiol. 2021 Dec 8;12:744437. doi: 10.3389/fmicb.2021.744437 (PMC8692253; doi:10.3389/fmicb.2021.744437)
Supplement: Supplementary file 1 [file Data_Sheet_1.docx]

Supplementary Material

**Supplementary Table 1.** List of abbreviations for Spirulina samples.

| Abbreviation | Sample treatments |
| --- | --- |
| WB | Wet spirulina biomass |
| WB_A_ | Wet spirulina biomass treated with Alcalase® |
| WB_F_ | Wet spirulina biomass fermented with *Lactiplantibacillus plantarum* T0A10 |
| WB_AF_ | Wet spirulina biomass treated with Alcalase® and fermented with *Lactiplantibacillus plantarum* T0A10 |
| DB | Dried spirulina biomass |
| DB_A_ | Dried spirulina biomass treated with Alcalase® |
| DB_F_ | Dried spirulina biomass fermented with *Lactiplantibacillus plantarum* T0A10 |
| DB_AF_ | Dried spirulina biomass treated with Alcalase® and fermented with *Lactiplantibacillus plantarum* T0A10 |
| LB | Lyophilized spirulina biomass |
| LB_A_ | Lyophilized spirulina biomass treated with Alcalase® |
| LB_F_ | Lyophilized spirulina biomass fermented with *Lactiplantibacillus plantarum* T0A10 |
| LB_AF_ | Lyophilized spirulina biomass treated with Alcalase® and fermented with *Lactiplantibacillus plantarum* T0A10 |

**Supplementary Table 2.** Area, number of peaks and peaks area of peptides as determined by Reversed-Phase Fast Performance Liquid Chromatography (RP-FPLC). Peaks area of detected peptides, expressed as percentage of the total area, is divided based on Eluent B percentage. All biomasses were also treated with Alcalase® (A, 1% vol/wt), fermented with *Lactiplantibacillus plantarum* T0A10 (F, final cell density of *circa* 7 log10 cfu/g) or fermented after enzymatic treatment (AF). Enzymatic and microbial treatments were carried out at 30 °C for 24 h.

|  |  |  | Peaks area (%) | | | |
| --- | --- | --- | --- | --- | --- | --- |
|  | **Area (mAU*ml)** | **N. peaks** | **0 %B** | **0-46 %B** | **46-100 %B** | **100 %B** |
| *Wet biomass* | | | | | | |
| WB | 1014 ± 42^f^ | 48 | 36.8 ± 1.5^d^ | 13.4 ± 0.5^e^ | 15.5 ± 0.6^b^ | 34.1 ± 1.3^b^ |
| WB_A_ | 1505 ± 58^e^ | 42 | 57.3 ± 1.9^ab^ | 20.9 ± 0.7^c^ | 3.3 ± 0.1^g^ | 18.2 ± 0.6^d^ |
| WB_F_ | 1587 ± 61^e^ | 36 | 51.9 ± 2.0^b^ | 20.1 ± 0.7^c^ | 4.4 ± 0.2^f^ | 23.3 ± 0.9^c^ |
| WB_AF_ | 1728 ± 65^d^ | 36 | 51.2 ± 1.8^b^ | 27.6 ± 0.9^b^ | 4.0 ± 0.1^f^ | 17.0 ± 0.5^d^ |
| *Dried biomass* | | | | | | |
| DB | 1087 ± 41^f^ | 26 | 12.0 ± 0.3^e^ | 11.9 ± 0.4^e^ | 22.4 ± 0.8^a^ | 53.5 ± 2.1^a^ |
| DB_A_ | 1071 ± 44^f^ | 34 | 57.9 ± 2.0^ab^ | 12.1 ± 0.3^e^ | 3.9 ± 0.3^f^ | 25.7 ± 0.6^c^ |
| DB_F_ | 949 ± 39^g^ | 35 | 35.9 ± 1.4^d^ | 16.9 ± 0.6^d^ | 13.5 ± 0.7^bc^ | 33.5 ± 1.3^b^ |
| DB_AF_ | 1478 ± 69^d^ | 40 | 43.2 ± 1.9^cd^ | 26.6 ± 1.0^b^ | 12.7 ± 0.2^c^ | 17.5 ± 0.8^d^ |
| *Lyophilized biomass* | | | | | | |
| LB | 4705 ± 183^c^ | 40 | 47.6 ± 1.9^c^ | 32.2 ± 1.2^a^ | 13.0 ± 0.5^c^ | 7.0 ± 0.3^e^ |
| LB_A_ | 7039 ± 259^a^ | 33 | 61.9 ± 2.3^a^ | 27.2 ± 0.8^b^ | 5.9 ± 0.3^e^ | 4.9 ± 0.1^f^ |
| LB_F_ | 4994 ± 205^d^ | 33 | 53.8 ± 2.1^b^ | 31.6 ± 1.3^a^ | 7.9 ± 0.4^d^ | 6.5 ± 0.4^ef^ |
| LB_AF_ | 6094 ± 236^b^ | 34 | 66.7 ± 2.4^a^ | 21.3 ± 0.8^c^ | 6.3 ± 0.3^de^ | 5.5 ± 0.3^f^ |

Data are the means of three independent analyses. ^a-h^Values in the same column with different superscript letters differ significantly (P < 0.05).

**Supplementary Figure 1.** Flow diagram of the experimental plan for Spirulina bioprocessing and analyses.
